# Innominate Artery Compression Syndrome: A Real Entity

**DOI:** 10.1016/j.atssr.2023.11.003

**Published:** 2023-11-22

**Authors:** Timothy W. Pettitt, Matthew Nungesser, Adele K. Evans

**Affiliations:** 1 Division of Pediatric Cardiothoracic Surgery, Children’s Hospital New Orleans, LCMC Health, New Orleans, Louisiana; 2 Division of Pediatric Otolaryngology, Children’s Hospital New Orleans, LCMC Health, New Orleans, Louisiana; 3 Department of Surgery, Louisiana State University Health Science Center, New Orleans, Louisiana

## Abstract

Extrinsic compromise of the lower airway in infants and children is most often due to vascular compression. Anterior tracheal compression caused by an aberrant course of the innominate artery is commonly referred to as innominate artery compression syndrome. We present a case of innominate artery compression syndrome causing severe tracheal compression precluding tracheostomy decannulation in a 6-year-old child who underwent previous neonatal repair of a left-sided congenital diaphragmatic hernia and coarctation of the aorta followed by tracheostomy and ventilator-dependent management of pulmonary hypoplasia and tracheobronchomalacia. Innominate artery translocation combined with aortopexy afforded adequate decompression without tracheoplasty for decannulation.

Innominate artery compression syndrome (IACS) is a common cause of airway compression in infants and children; however, most are asymptomatic. Perhaps this explains the hesitancy of some surgeons to treat this as a real entity that requires surgical intervention. Congenital diaphragmatic hernia (CDH) is often associated with cardiovascular malformations but, to our knowledge, not IACS. We hypothesize that the mediastinal shift typically seen in patients with CDH and unilateral lung hypoplasia provides the substrate for IACS to develop. Surgical repair typically involves either aortopexy or reimplantation of the innominate artery. Combining the 2, using intraoperative bronchoscopy to guide the repair, would appear to be beneficial and to optimize outcomes.

This is a male patient born at 38 3/7 weeks with prenatal diagnosis of left-sided CDH. The patient underwent repair of the CDH on day 2 of life. He additionally underwent repair of coarctation by an extended end-to-end anastomosis through left thoracotomy at 2 weeks of life. Given the significant degree of left lung hypoplasia and chronic respiratory failure, the patient required tracheostomy for long-term home ventilation.

At 4 years old, he had achieved independence from mechanical ventilation and presented again for consideration of decannulation. He underwent 3 separate speaking-valve (Passy Muir) trials, which resulted in immediate pallor and dyspnea. He underwent operative sleep endoscopy and rigid endoscopy, which demonstrated 80% compression of the midtracheal lumen by an anterior, pulsating external force that dynamically worsened to nearly complete obstruction during respiratory cycling. Computed tomography angiography (CTA) of the chest demonstrated leftward mediastinal shift with hyperexpansion of the right lung across the midline into the left side of the chest. There was severe narrowing of the midtrachea at the level where the innominate artery crossed anteriorly (Figure 1).

**Figure 1:**
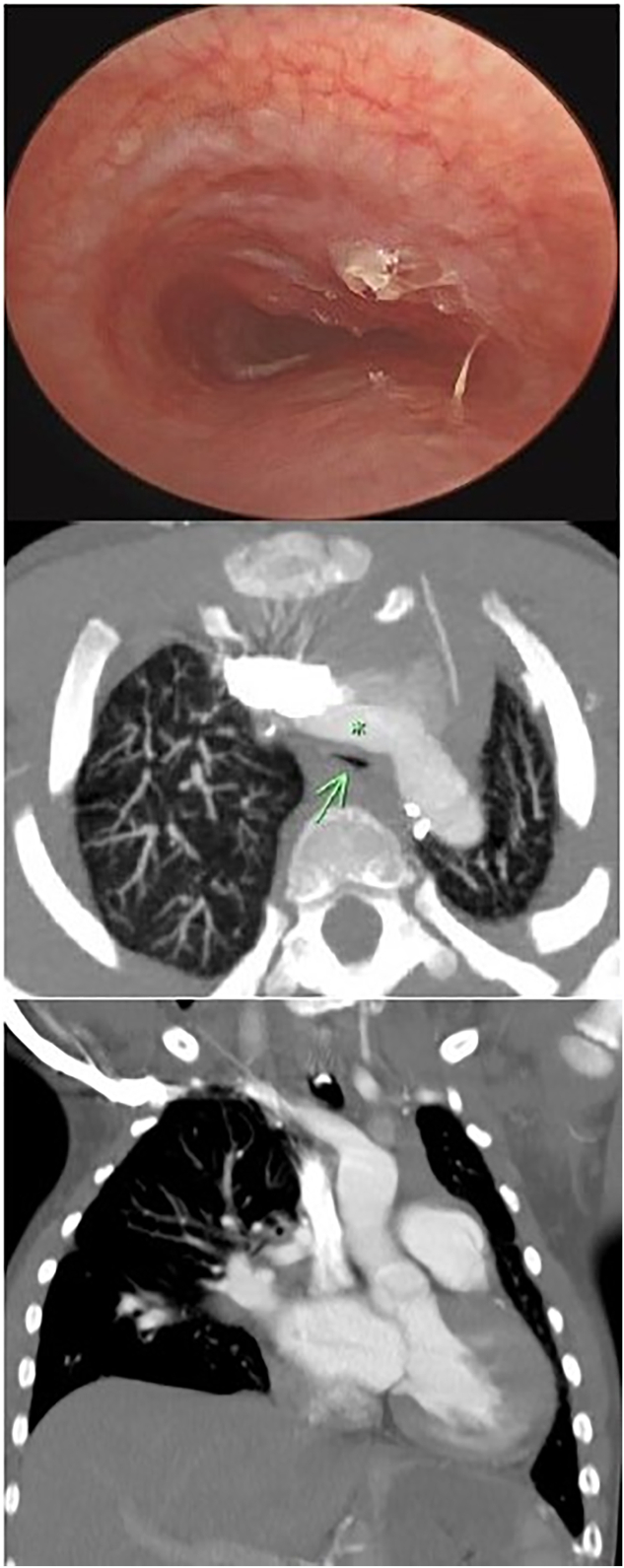
Preoperative bronchoscopy and computed tomography angiography demonstrating severe compression of trachea (arrow) by innominate artery (asterisk).

The patient was referred for surgical intervention at 6 years of age. A median sternotomy was performed. The right lobe of the thymus was excised. After heparinization, the innominate artery was clamped distally, controlled proximally with a partial occluding clamp on the aorta, and transected at its origin. The resulting opening in the aorta was then cut back to a more proximal and anterior position by 1.5 cm. The original, more distal opening was oversewn with 5-0 Prolene suture. Considering his anatomy and anticipating that translocation alone may not be enough to maximally relieve the compression of the trachea, it was decided to proceed with an aortopexy to further distract the position of the innominate artery from the trachea. Risk of compression of the innominate vein during fixation of the innominate artery to the sternum prevented the degree of aortopexy believed to be warranted. Therefore, the innominate vein was relocated posterior to the innominate artery, removing that risk. The innominate artery was reanastomosed to the new position on the aorta. The neoinnominate-aortic junction was then firmly fixated to the left hemisternum with several pledgeted, nonabsorbable sutures (Figure 2). Manipulation of the innominate artery was guided by intraoperative flexible bronchoscopy, which revealed remarkable improvement in airway patency with minimal residual luminal deformation. Postoperatively, the patient demonstrated dramatic improvement in language skills, exercise tolerance, and energy. Follow-up bronchoscopy and CTA demonstrated a widely patent trachea with wide separation of the innominate artery from trachea (Figure 3). At 3 months postoperatively, he was no longer dependent on his tracheostomy, findings of a capped sleep study were normal, and he was successfully decannulated.

**Figure 2:**
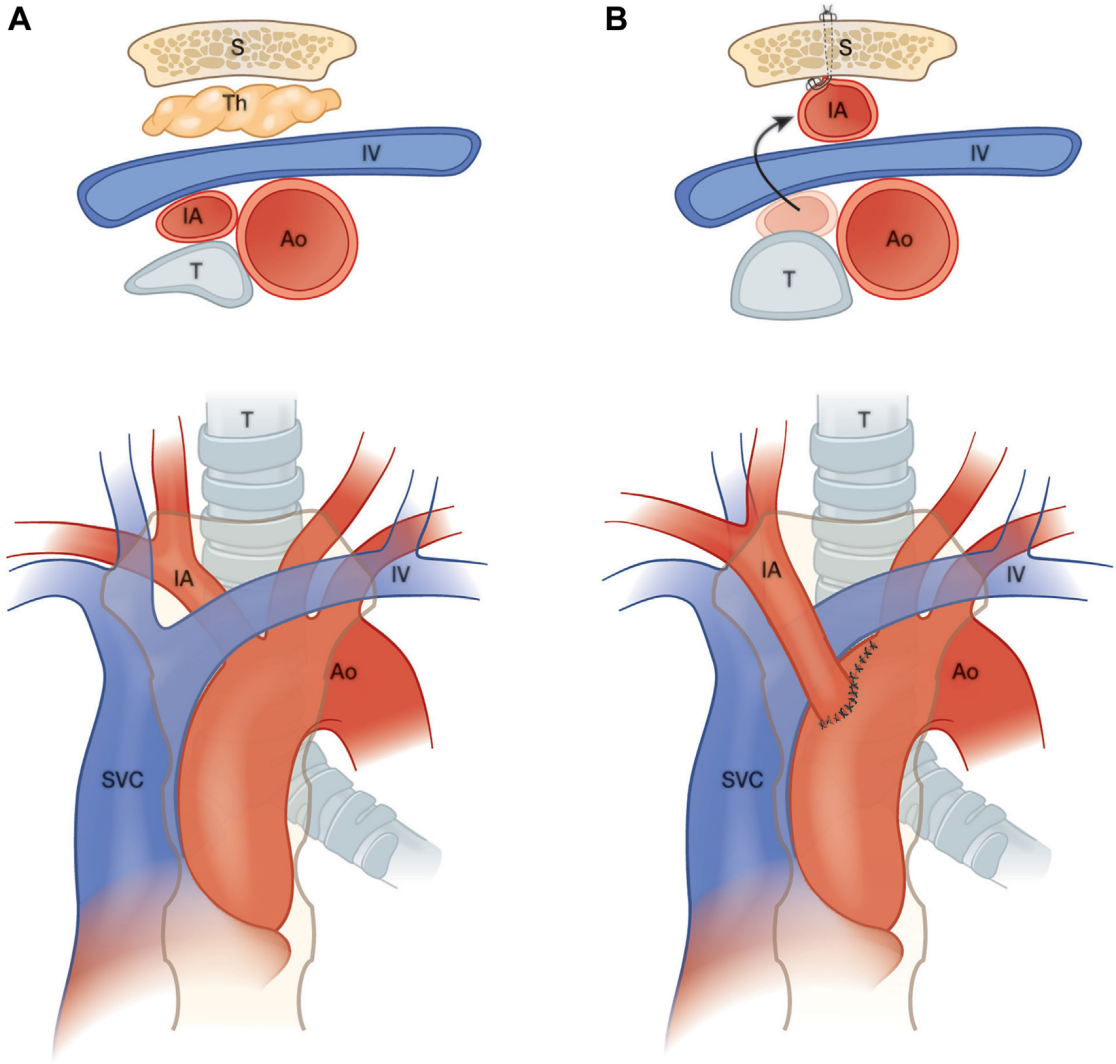
(A) Preoperative and (B) postoperative illustration demonstrating axial and coronal views of anatomy. Translocation of innominate artery denoted by arrow. (Ao, aorta; IA, innominate artery; IV, innominate vein; S, sternum; SVC, superior vena cava; T, trachea; Th, thymus.)

**Figure 3:**
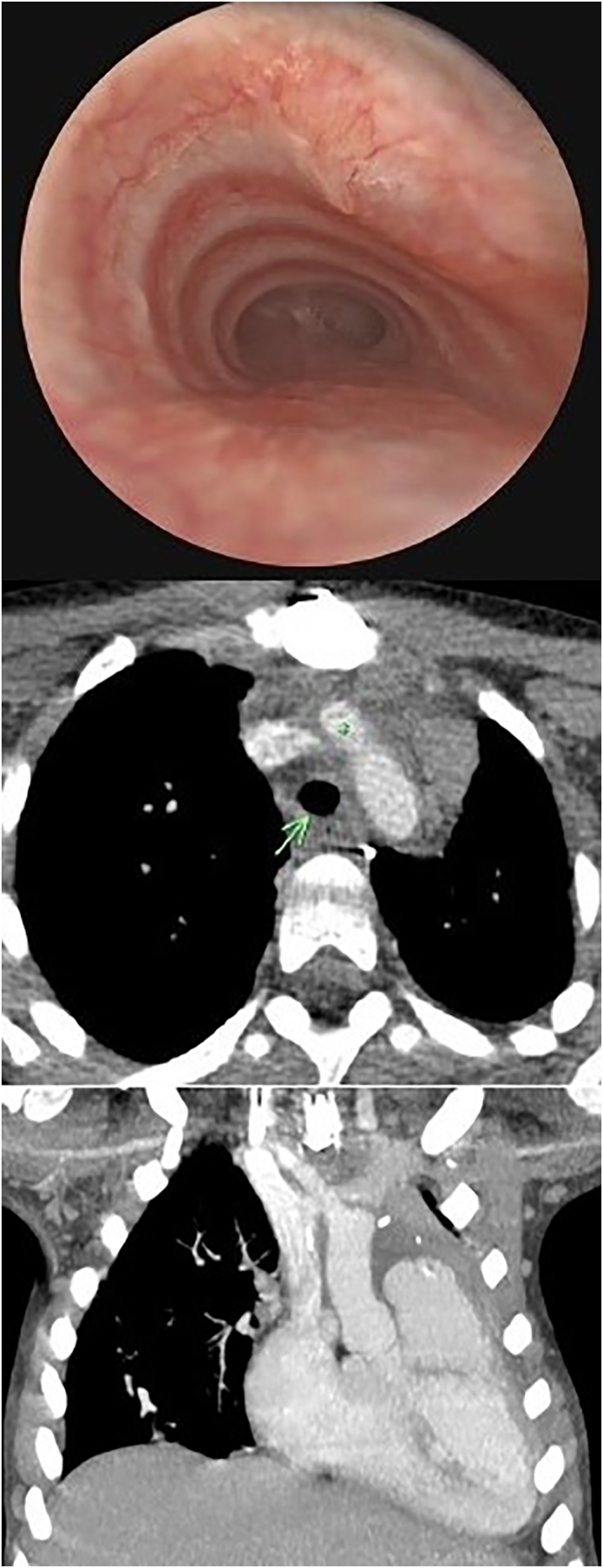
Postoperative bronchoscopy and computed tomography angiography demonstrating widely patent airway with wide separation of the innominate artery (asterisk) from trachea (arrow).

## Comment

IACS has previously been described in an infant with CDH and theorized to develop because of leftward mediastinal shift from unequal expansion of the lungs, which in turn affects the growth and course of the innominate artery as it traverses the mediastinum.^1^ One can speculate that hypoplasia of the left lung promotes a leftward shift of the mediastinum and aortic arch. As the arch deviates more into the left side of the chest, it is drawn posteriorly along the spine. This leftward and posterior deviation of the aorta–innominate artery junction brings the artery in closer apposition to the trachea and creates a “bowstring” effect across the airway.

Surgical repair of IACS typically involves either aortopexy^1^^,^^2^ or innominate artery reimplantation or translocation.^3^^,^^4^ Whereas both have high success rates, long-term follow-up studies demonstrate overall better outcomes with reimplantation compared with aortopexy.^4^ Potential drawbacks to aortopexy include weakening or dehiscence of the adventitial sutures fixating the aorta–innominate artery to the sternum, inadequate suspension of the artery due to the presence of a large thymus, and risk of entrapment of the left innominate vein between the aorta or innominate artery and sternum, leading to left arm swelling (T.W.P., personal experience). We believe thymic resection is a key component of either surgical approach to facilitate adequate suspension, in the case of aortopexy, or to give the innominate artery more room anteriorly, after reimplantation. We are surprised that there is not more discussion in the literature of the risk of left innominate vein entrapment with anterior aortopexy. The left innominate vein typically crosses anterior to the aorta–innominate artery junction and is at risk of entrapment during aortopexy.

Reimplantation, on the other hand, has the advantage of immediately correcting the substrate for IACS, which is the leftward, posterior deviation of the innominate artery origin. However, there is a limit to how far one can translocate the origin of the innominate artery unless one uses an interposition graft to lengthen the artery. Reimplantation of the innominate artery, with its obligatory division and translocation, permits the additional relocation of the innominate vein posterior to the artery and allows aggressive traction and fixation of the aorta–innominate artery junction to the sternum. To our knowledge, this combination of innominate artery reimplantation with relocation of the innominate vein posterior to the artery to facilitate optimal aortopexy has not been previously described in the literature. A retroarterial innominate vein is akin to a retroaortic left innominate vein, which is a naturally occurring variant. There was no left innominate venous obstruction on postoperative CTA or signs of left arm swelling on physical examination.

Our case supports the hypothesis that patients with CDH, especially left sided, are at an increased risk for development of IACS as a result of unequal lung development with shift in mediastinal and vascular structures. Patients with CDH with airway symptoms or difficulty with weaning from mechanical ventilation or tracheostomy decannulation should be evaluated for IACS by airway endoscopy and CTA, if indicated. Dramatic relief of airway compression by an anomalous innominate artery is achievable through median sternotomy with a combination of translocation of the origin of the artery to a more proximal and anterior position on the aorta and aortopexy. Relocation of the left innominate vein posterior to the translocated innominate artery can facilitate aortopexy of the aorta–innominate artery junction to the sternum, eliminating the risk of vein entrapment between the aorta and sternum, and maximize decompression of the airway. This patient’s response should leave little doubt that IACS is a real entity and is amenable to surgical intervention.

## References

[1] Yonekura T., Hirooka S., Kubota A. (2000). Surgical intervention for emphysematous pulmonary regions in a postoperative infant with congenital diaphragmatic hernia. J Pediatr Surg.

[2] Wine T.M., Colman K.L., Mehta D.K., Maguire R.C., Morell V.O., Simons J.P. (2013). Aortopexy for innominate artery tracheal compression in children. Otolaryngol Head Neck Surg.

[3] Hawkins J.A., Bailey W.W., Clark S.M. (1992). Innominate artery compression of the trachea. Treatment by reimplantation of the innominate artery. J Thorac Cardiovasc Surg.

[4] Grimmer J.F., Herway S., Hawkins J.A., Park A.H., Kouretas P.C. (2009). Long-term results of innominate artery reimplantation for tracheal compression. Arch Otolaryngol Head Neck Surg.

